# Moth versus fly: a preliminary study of the pollination mode of two species of endemic Asteraceae from St Helena (*Commidendrum robustum* and *C. rugosum*) and its conservation implications

**DOI:** 10.3897/BDJ.8.e52057

**Published:** 2020-05-06

**Authors:** Mikko Pasi Tapani Paajanen, Quentin Cronk

**Affiliations:** 1 University of British Columbia, Vancouver, Canada University of British Columbia Vancouver Canada

**Keywords:** endemic plants, conservation, interspecific hybridisation, gene flow, fragmented populations, myophily, phalaenophily

## Abstract

*Commidendrum robustum* (Roxb.) DC. (St Helena gumwood) and *C. rugosum* (Dryand.) DC. (St Helena scrubwood) are ecologically important, endemic woody Asteraceae from the isolated South Atlantic island of St Helena. Once very abundant, they now exist in sparse fragmented populations due to 500 years of environmental destruction. They are sister taxa that evolved on the island and are reported to hybridise. *Commidendrum rugosum* has a saucer-like erect capitulum, whereas *C. robustum* has a somewhat globular hanging capitulum. Using daytime timelapse photography to follow capitula through their life cycle, we found that *C. rugosum* appears to be myophilous, visited largely by flies (including the endemic syrphid, *Sphaerophoria beattiei* Doesburg & Doesburg) and occasionally by Lepidoptera. *Commidendrum robustum*, on the other hand, although visited by flies, strongly attracts moths (especially noted at the Millennium Forest site). Our data suggest that moth visits may reduce visits from flies due to the sensitivity of flies to interference by other insects. We conclude that *C. robustum* may have a mixed syndrome of myophily/phalaenophily and that there is apparently some divergence of the pollination niche between the two species. Its potential in attracting moths, coupled with its former abundance, suggests that it may have been a major food source for adults of the numerous endemic moths. Pollinator activity was measured by insect visitation rates (mean visits per capitulum per day, V) and insect residence time (mean pollinator kiloseconds per capitulum per day, R). Both are higher for *C. robustum* (*C. rugosum*, V = 16.4, R = 3.101; *C. robustum*, V = 34.0, R = 8.274), reflecting the abundance of moths on the capitula at the Millennium Forest site. The conservation implications of the pollination mode are that: (1) there is considerable pollinator activity on the capitula and pollination is not currently a limiting factor for plant reproduction; (2) gene exchange between geographically-isolated populations of *C. rugosum* is likely to be minimal due to the apparent reliance of the species for pollination on small flies (especially *Sphaerophoria beattiei*), which are believed to be not effective as pollinators over long distances (> 1 km). A possible exception is the strong-flying drone-fly, *Eristalis tenax* Linn. which, although not as abundant as *Sphaerophoria*, does visit the flowers; (3) there is considerable overlap between the two species in flower visitors and interspecific pollen transfer is possible where the two species grow intermixed (which has potential positive and negative implications for species survival).

## Introduction

St Helena, an isolated Miocene volcanic oceanic island of 121.7 km^2^, has a remarkable endemic flora (Ashmole and Ashmole 2000, Cronk and Ninnes 2000). Amongst these endemics are four species of “tree daisies” in the genus *Commidendrum* (Asteraceae) which, together with the monotypic sister genus *Melanodendron*, provide an interesting example of adaptive radiation in plants on St Helena (Eastwood et al. 2004). *Commidendrum* and *Melanodendron* species between them contributed greatly to all the major pre-colonisation vegetation types of St Helena, from coastal semi-deserts to the cloud forests of the Central Peaks (Cronk 1989).

**Figure 1.**
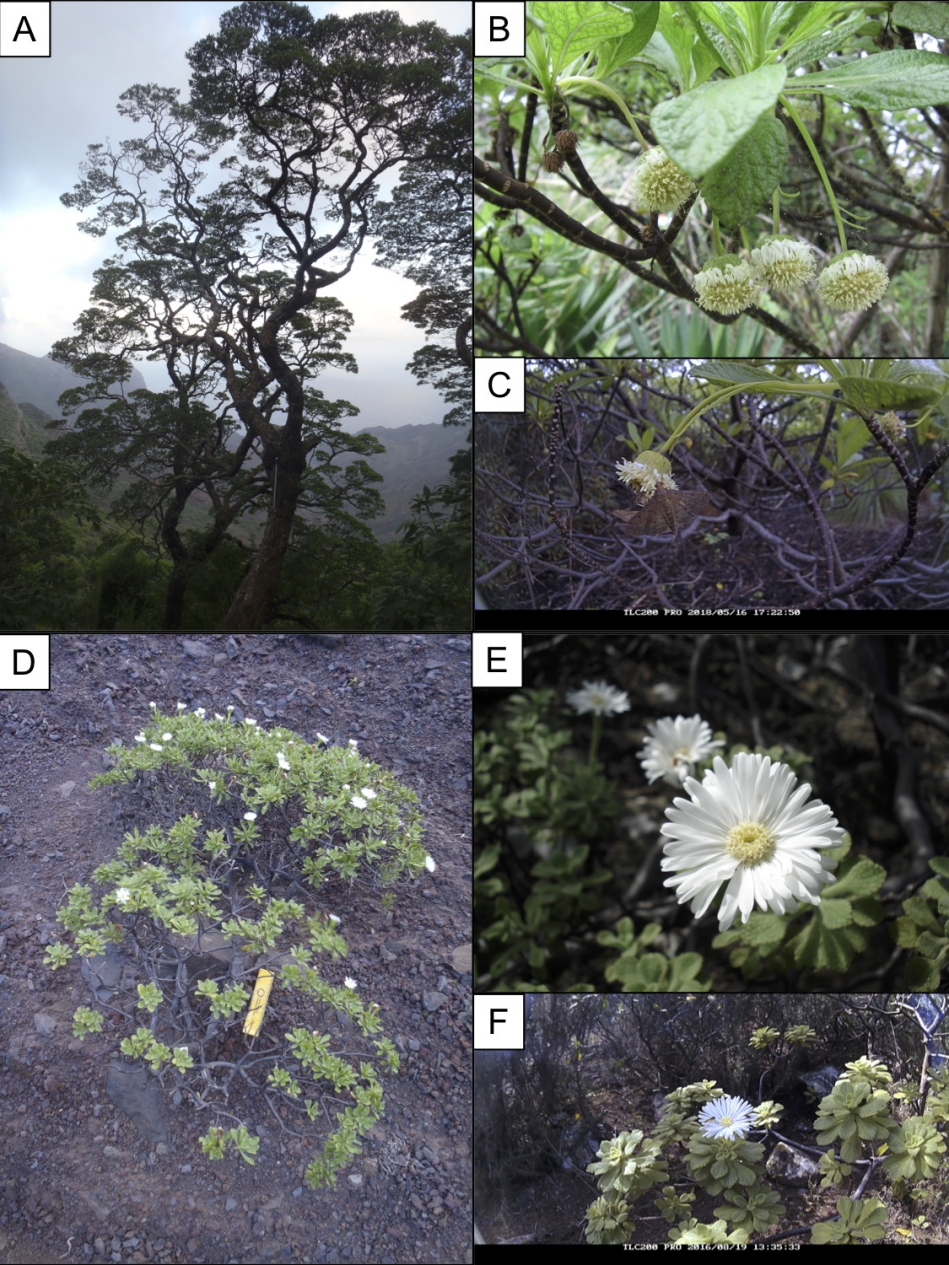
General form (A, D) and capitula (B, E) of St Helena gumwood (*Commidendrum robustum*) and St Helena scrubwood (*C. rugosum*), respectively. Flower visitors to gumwood (C) and scrubwood (F) capitula.

*Commidendrum robustum* (Roxb.) DC. (St Helena gumwood) and *C. rugosum* (Dryand.) DC. (St Helena scrubwood) are closely related (Eastwood et al. 2004), but morphologically and ecologically clearly distinct species (Fig. 1), the former being a middle-sized tree occupying dry and moist forests in the intermediate elevations and the latter one being a small to middle-sized shrub occupying dry semi-deserts and inland cliff areas (Ashmole and Ashmole 2000, Cronk and Ninnes 2000, Lambdon 2012, Cronk 1989). These two species are known to hybridise with each other in areas where they are growing in sympatry, forming morphotypes intermediate to both of the parental species (Eastwood et al. 2004).

Ecological speciation is believed to have played an important role in the divergence of *Commidendrum* lineages from each other (Eastwood et al. 2004). There are marked differences in the capitulum morphology between the sister species *C. robustum* and *C. rugosum*: hanging capitula with small ray florets for the former and upward facing capitula with showy ray florets for the latter (Fig. 1). These differences suggest the possibility that differences in pollinators may have enforced the ecological separation of these hybridising species as has been suggested in other systems (Van der Niet et al. 2014). In addition to the differences in overall capitulum morphology, there are also differences in disc flower morphology. The gumwood anther tubes extend to around twice the length outside of the mouth of the floret compared to those in scrubwood (1.8 vs. 0.9 mm). This affects the height above the corolla tube, at which the pollen is presented and at which pollination occurs.

**Figure 2.**
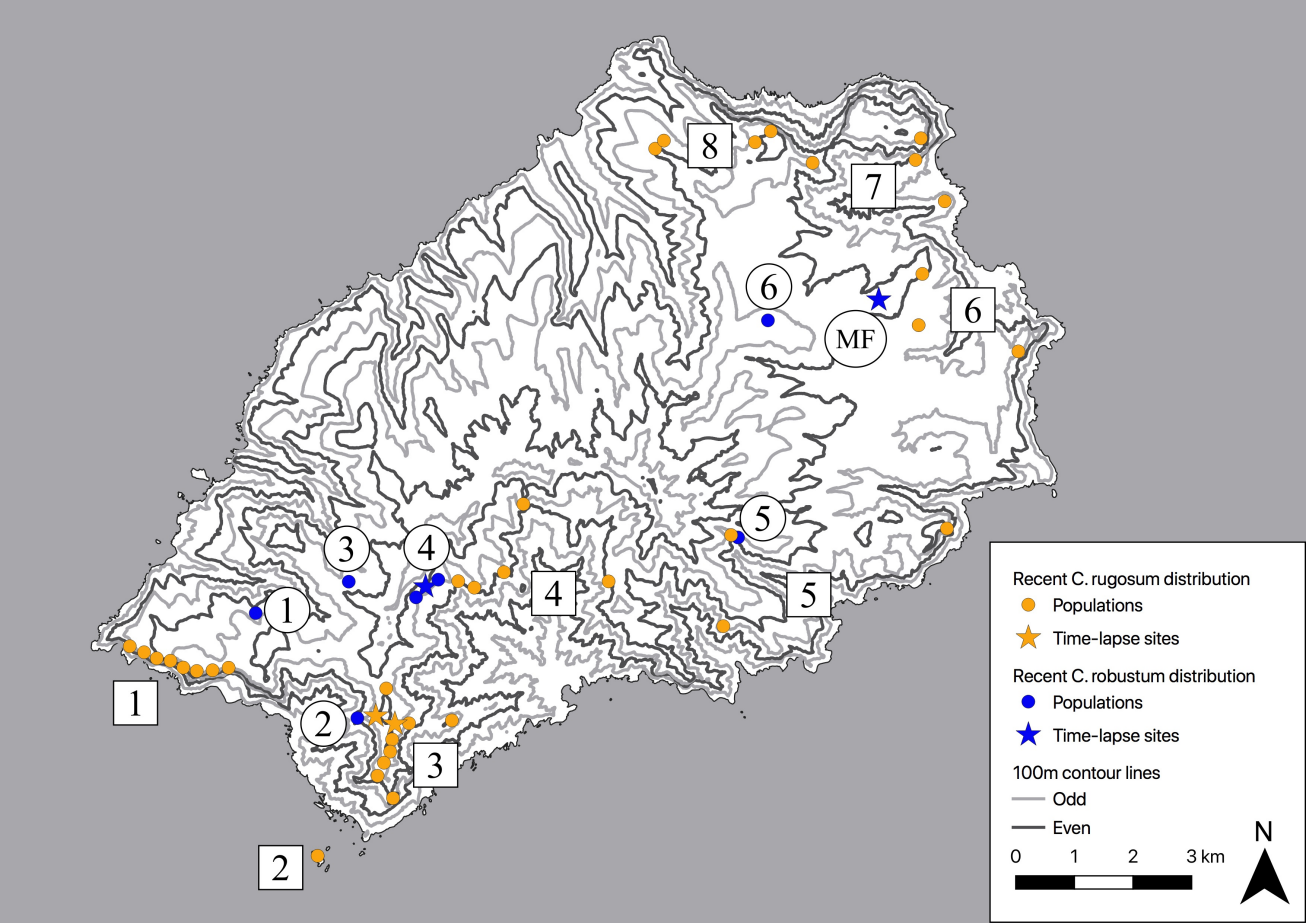
Map of recent (extant and < 50 yrs ago) *Commidendrum robustum* and *C. rugosum* populations on St Helena. Key to *C. rugosum* populations (square): 1. Man & Horse cliffs, 2. Speery Island, 3. Blue Point area, 4. Sandy Bay inland cliffs, 5. Eastern valleys, 6. Prosperous Bay Plain area, 7. Turk’s Cap and the Barn, 8. Flagstaff and Pipe Ridge area. Key to *C. robustum* populations (round): 1. Man & Horse (extinct), 2. Devil’s Cap Ridge (extinct), 3. Thompson’s Wood, 4. Peak Dale, 5. Deep Valley, 6. Piccolo Hill, Longwood. Note that two of these populations are marked as recently extinct. The former distribution (> 100 yrs ago) was considerably more extensive. The Millennium Forest gumwood site (planted) is also indicated (MF). Other planted sites are not shown.

Due to the small geographical size of St Helena, these two species have always been growing relatively close to each other (Fig. 2), especially the inland cliff populations of *C. rugosum*, which would have been surrounded by forests of *C. robustum* (Cronk 1989). There may have been selection against hybridisation, since the hybrid individuals are likely to be less well adapted to the environments of either parental species (e.g. smaller and slower growing than *C. robustum* and less drought tolerant than *C. rugosum)*.

Pollination mode (defined here as the type, mechanism and effectiveness of pollination) is important basic data for any species conservation programme. As pollinator-mediated seed set and gene flow in rare island endemic plants is relevant to many aspects of species biology (e.g. successful seed reproduction, genetic diversity, hybridisation and evolution) and hence to conservation and extinction, our aim in this study was to characterise, in a quantitative manner, insect visitation to the capitula of the two species. For this, we used time lapse photography, a relatively new and underused method for recording invertebrate visitation on flowers (Edwards et al. 2015). More specifically, we wished to answer the questions of the identity of floral visitors to these capitula, the number and duration of visits, potential differences and similarities between the plant species and whether these factors might facilitate (or inhibit) not only seed set, but also genetic connectivity and interspecific hybridisation. We note that this study is preliminary in that we were not able to perform a complete survey of seasons and localities due to time and logistical constraints. Nevertheless, these data represent the first detailed analyses of the pollination of a St Helena endemic and they provide clear evidence of species-specific differences.

## Material and methods

**Figure 3.**
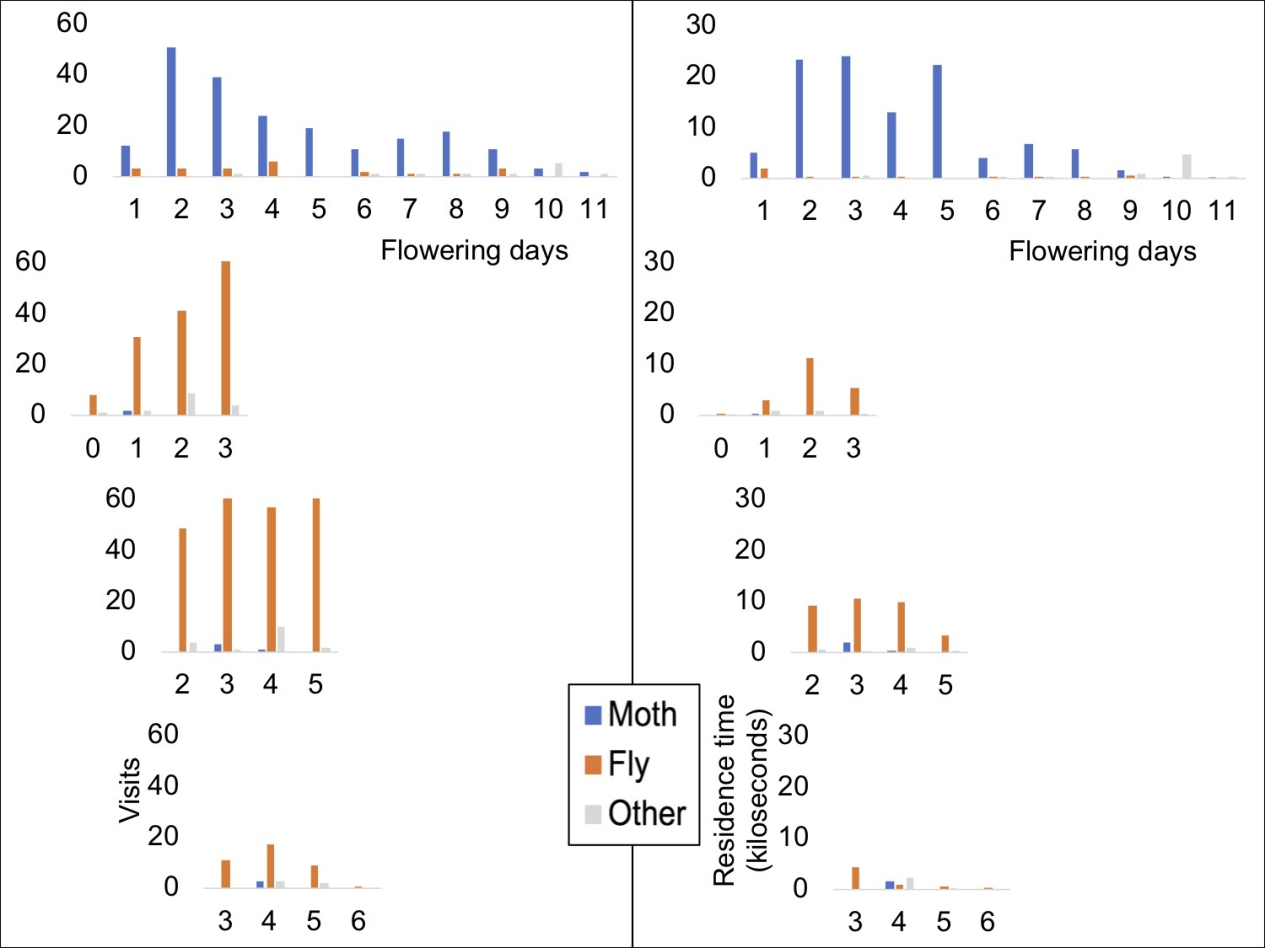
Visitation frequency (left) and time on *Commidendrum robustum* capitula (right) in Millennium Forest (uppermost) and Peak Dale (rest). Peak Dale capitula were followed for four days with early, middle and late stage of flowering capitula represented in the three graphs, respectively.

**Figure 4.**
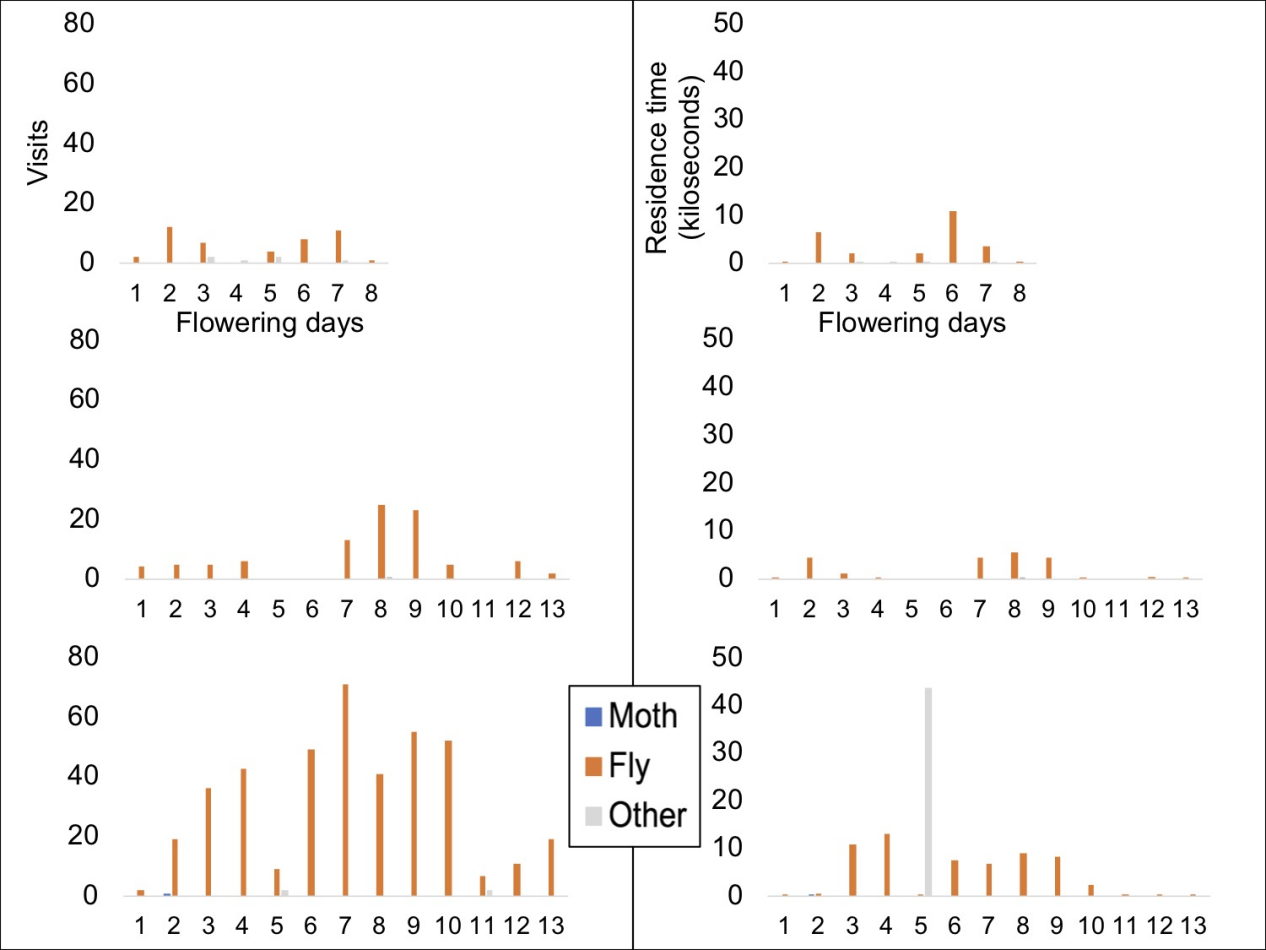
*Commidendrum rugosum* flower visitation frequency (left) and residence time (right). All three capitula were at Blue Point, upper two on the windward side of the ridge and the third one on the leeward side of the ridge.

Time lapse footage was taken on St Helena using a *Brinno TLC200 pro* time lapse camera (Edwards et al. 2015). Sites used for recording are shown in Fig. 2. One *Commidendrum robustum* capitulum was followed in the Millennium Forest for 11 days (24 May – 3 June) in 2018, which covered the whole active flowering period of the capitulum. Additionally, a branch of several capitula in Peak Dale was followed for 4 days (16–19 January), in 2017, out of which three capitula were chosen for detailed observations and so provide an estimated whole active flowering period for a capitulum in the observed tree. For *C. rugosum*, three capitula were followed for 8, 13 and 13 days in Blue Point in 2016 (7–14 June, 2–14 July and 13–15 August, respectively) (Figs 3, 4). Two of the capitula (both 13 days) were followed through the whole estimated active flowering period of the capitulum (approximately 10 days). Photographs were generally taken every 3 seconds (Edwards et al. 2015). The camera was set to record approximately the full diurnal period from dawn to dusk (i.e. 5.30 am to 7.00 pm). At Millennium Forest, photographs were taken every 5 seconds for more efficient use of battery life, but this is unlikely to make a major difference to the data collected. In addition to the daytime timelapse observations, field observations were made of night-time pollinators. No observations were made on diurnal patterns of nectar flow in the flowers, but this would be a useful subject for further study.

Time lapse footage was reviewed, flower visitors identified to species (most main visitors) or morphotaxa and the duration of each visit recorded and graphed. Duration of visit was recorded from first photographic record to last photographic record and it is thus a conservative measure (minimum duration). Insects in a single frame were recorded for presence, but received no duration. For graphing purposes, insects were pooled into taxonomic groups (order or suborder). Figures for pollinator activities include all most likely potential pollinators, i.e. Lepidoptera, Diptera and Hymenoptera, but exclude beetles, spiders and other arthropods, which were minor visitors and considered less likely to be effective pollinators for the two *Commidendrum* species. The original data are available on Figshare (https://doi.org/10.6084/m9.figshare.11971884.v1).

### Site details

Time lapse imagery was recorded in three places on St Helena, *Commidendrum robustum* capitula in Millennium Forest and Peak Dale and *C. rugosum* capitula in Blue Point (Fig. 2). All capitula recorded were 30-50 cm from the ground level, due to the height of the camera and tripod used. The Millennium Forest capitulum was situated at 430 m of elevation, approximately 20 m from the edge of a dense *C. robustum* planting, on a low branch of a young tree of 2 m high. Millennium Forest is the largest individual ecological restoration site on St Helena, with varied plantings of dryland endemic species. *C. robustum* has not generally reached over 3 m high in this seasonally very dry and exposed site. Peak Dale capitula were situated in 560 m of elevation, approximately 15 m from the edge of the *C. robustum* forest, on a low branch of a tree. Peak Dale is the only remaining relatively sizeable natural stand of *C. robustum* forest, with trees reaching to 10-15 m in height and thus most of the capitula higher up than the ones recorded with the time-lapse camera. Blue Point capitula were situated in 560 m of elevation on the windward side of the ridge and 580 m of elevation on the leeward side of the ridge, on *C. rugosum* shrubs less than 1 m high. The windward side of the Blue Point ridge is highly exposed, with low and sparse vegetation and a relatively large *C. rugosum* population. The leeward side is dominated by introduced thicket, up to 5 m high, with a small *C. rugosum* population situated in a few openings in the higher vegetation. Blue Point is one of the strongholds of endemic dryland vegetation on St Helena.

## Results

### General patterns of visit

**Figure 5.**
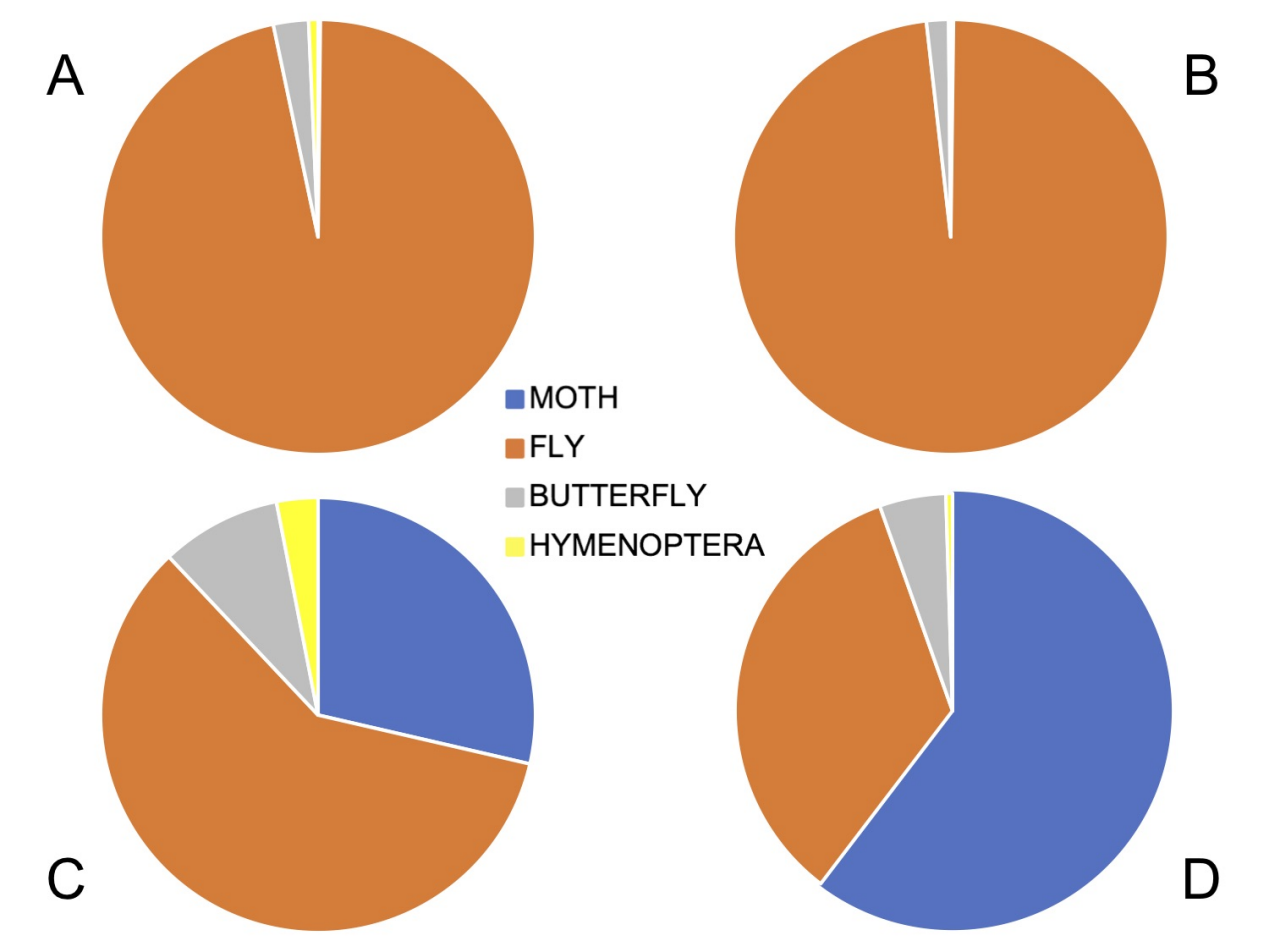
Pie charts to illustrate pollinator preference, in visits (A, C) and residence time (B, D) for *Commidendrum rugosum* (A, B) and *C. robustum* (C, D). Proportions for moths, flies, hymenoptera and butterflies (Lycaenidae).

According to the time-lapse data, the main diurnal visitors are Lepidoptera and Diptera (Table 1) with Lepidoptera tending to occur more often on *Commidendrum robustum* and Diptera characteristic of *C. rugosum*, but also visiting C. robustum frequently. The main Diptera pollinators are the endemic Loveridge’s hoverfly *Sphaerophoria beattiei*, the non-endemic hoverfly *Syritta stigmatica* and the “small black fly” morphotaxon (possibly more than one species of very small Diptera: Muscidae/Anthomyiidae and possibly including the endemic muscid *Limnophora helenae*). (Unfortunately this morphotaxon is too small for critical identification features to be resolved by our camera system. Further studies will be needed to identify this/these species). The main Lepidoptera pollinators are the introduced moths *Spoladea recurvalis* and *Herpetogramma licarsisalis* and the non-endemic long-tailed blue butterfly *Lampides boeticus*. Florets in the capitulum open sequentially over several days (Table 2;Suppl. material 1). The peak visits occurred when the majority of florets were open during mid-flowering of the capitulum (Figs 3, 4). Our data do not include nocturnal visitors, but general field observations of gumwood indicate that nocturnal visits by several moth species occurs. Night-vision equipment was not available to record this, but full 24 hour recordings would be of interest for future work. Combined data on pollinator preferences are shown graphically in Fig. 5.

**Figure 6.**
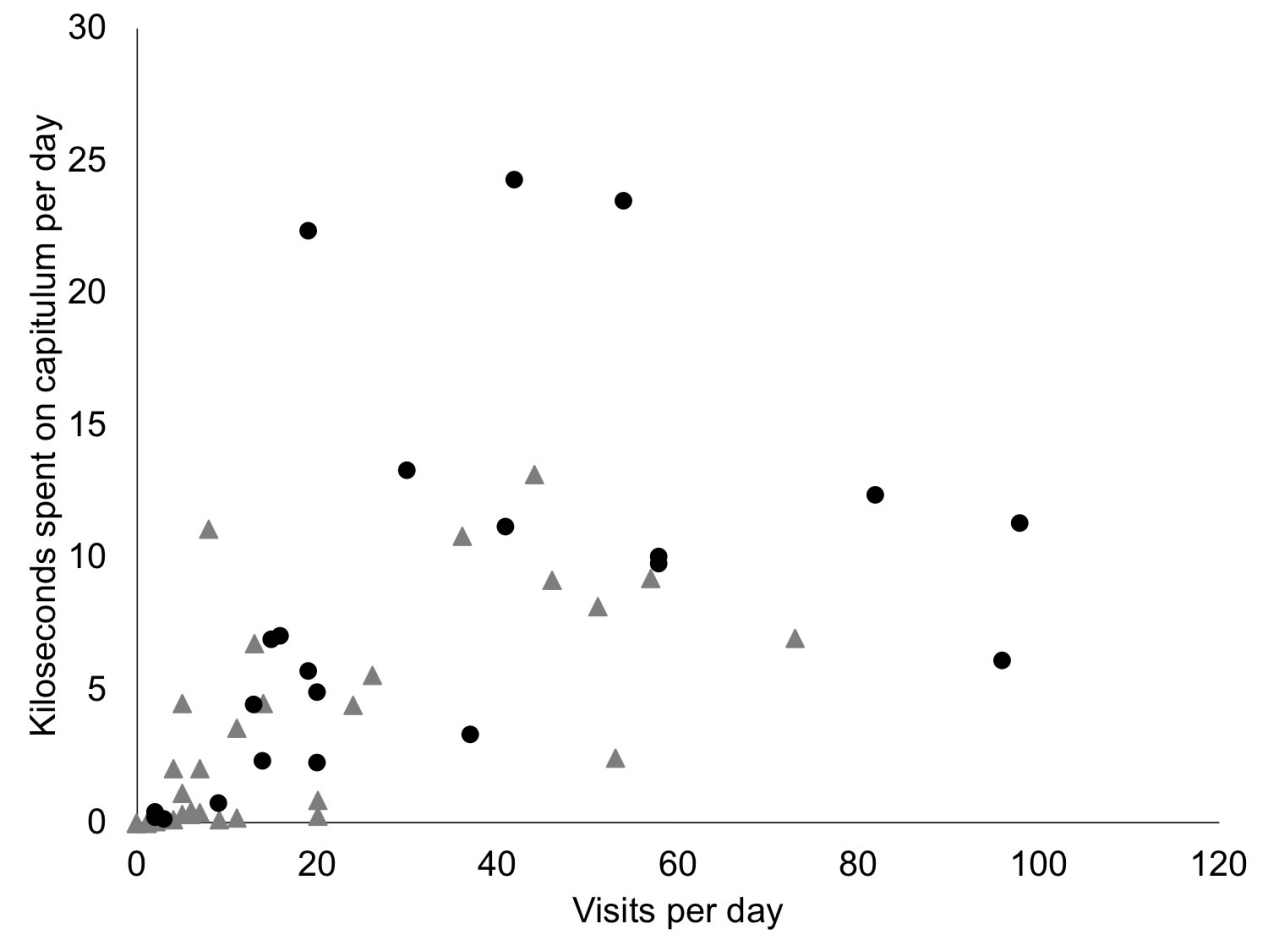
Daily totals of pollinator activity per capitulum, with total number of visits per day plotted against total pollinator time spent on a capitulum. Each dot represents a capitulum on a particular day. Pollinator activity varies between good days and poor days depending mainly on weather (wind and rain). Grey triangles = *Commidendrum rugosum*, mainly flies. Black dots = *C. robustum*), moths and flies.

Overall, there is considerable pollinator activity in both the species (Fig. 6), whether measured as insect visitation rates (mean/median visits per capitulum per day, V) or insect residence time (mean/median pollinator kiloseconds per capitulum per day, R). Differences in pollinator type (moth vs. fly) affect patterns of pollinator activity. For *C. rugosum* (mainly fly), the measures are: V = 16.4 and R = 3.101 (medians V = 7, R = 0.864) and for *C. robustum* (mainly moth), they are: V = 34.0 and R = 8.274 (medians V = 20, R = 6.488). It is evident that moth visitors tend to be on *C. robustum* and spend longer on a visit than flies (mean visit duration: moth 505 seconds; fly 189 seconds).

Finally, we noted that moths and syrphid flies interact differently with capitulum morphology. *Commidendrum robustum* has longer projecting anther tubes, ca. 1.8 mm versus 0.9 mm in *C. rugosum*. Moths alight on the tops of the projecting styles and anther tubes of *C. robustum* and may be seen to probe the corolla tubes from above with their probosces.

### Flower visitation on *Commidendrum robustum*

Patterns of visits to individual *C. robustum* capitula are shown in Fig. 3. At the Millennium Forest site, the main visitors recorded were two introduced species of day-flying moths, *Spoladea recurvalis* and *Herpetogramma licarsisalis* (Fig. 3). Hoverflies were also recorded from the capitulum, *Syritta stigmatica* being the main species. The endemic hoverfly *Sphaerophoria beattiei* was not recorded from the Millennium Forest capitulum. Peak Dale capitula were visited mainly by Diptera, especially *Sphaerophoria beattiei*. Interestingly, day-flying moths were rare on Peak Dale capitula, the main day-time Lepidoptera visitor being the non-endemic butterfly *Lampides boeticus*. Although no quantitative night-time observations were made, general field observations revealed a number of moths visiting *C. robustum* capitula after dark at both sites. Identified nocturnal species at Millennium forest included numerous introduced moths: *Herpetogramma licarsisalis*, *Hypocala rostrata* (Fabricius, 1794), *Spodoptera littoralis* (Boisduval, 1833), *Spoladea recurvalis* and *Trichoplusia ni* (Hübner, 1803). At Peak Dale (21 February 2014), the endemic moth *Helenoscoparia scintillulalis* (Wollaston, 1879) was observed, as well as the introduced *Hypocala rostrata* (Suppl. material 2).

### Flower visitation on *Commidendrum rugosum*

Patterns of visits to individual *C. rugosum* capitula are shown in Fig. 4A–F. In general, it seems that scrubwood capitula are mainly visited by Diptera, although a non-endemic butterfly, *Lampides boeticus*, is also a relatively common visitor. On capitulum 6, it is noticeable how on day 5 very few flies visited the flower while there is a high level of visitation on other capitula. We attribute this to the presence of a spider (marked as “other” in the figures) that occupied the capitulum for most of the daylight hours. Interference between arthropod visitors is thus an important factor in pollination. Weather conditions also contribute considerably to the abundance of flower visitors, especially on exposed sites. This can be seen on capitulum 3 on day 4 and on capitulum 4 on days 6 and 7, all of which were very windy and/or rainy with no visitors to the capitulum. Field observations at night failed to find moths or any other nocturnal pollinating insects on *C. rugosum* capitula.

### Insect-insect interaction and pollinator interference

The absence of insects on a capitulum occupied by a spider (noted above) is an example of an arthropod interaction affecting pollination. However, we were also interested in whether there were interactions between pollinator insects (particularly between moths and flies) that could affect pollination mode. To gain evidence about this, we first looked at patterns of “double occupancy”, cases in which two insects shared the same capitulum. We found this to be relatively rare, including 77 instances of moth-moth double occupancy (all on *C. robustum*), resulting from a total number of moth visits recorded in our study of 215 (35.8%). This compared with only 22 cases of fly-fly double occupancy (20 in *C. rugosum*, two in *C. robustum*), resulting from a total of 997 fly visits (2.2%). Most of these cases (14) involved the “small black fly” morphotaxon alone, rather than syrphids. In only two instances did we find two syrphids sharing a capitulum. There was only one case of moth-fly double occupancy (involving the “small black fly”).

To further investigate the possible sensitivity of flies to other insects, of larger or smaller size, we examined instances of “replacement”, where one insect is immediately replaced by another in a timelapse series, indicating the possibility of an interaction between the two. We found 19 replacements involving flies. There were 12 instances of fly replacing fly, six cases of which were of a larger fly replacing a smaller, two cases of a smaller fly replacing a larger and four same size replacements. There were seven instances of flies being replaced by non-fly flower visitors, in all cases the other visitors were larger in size. Conversely, we found no instances of flies replacing non-flies. Although the numbers are small, the patterns are consistent with flies (especially syrphids) not being attracted to capitula occupied by other organisms and, if on a capitulum, being readily disturbed by other organisms (particularly if larger than themselves).

## Discussion

### Evidence for differentiation in pollination niche between gumwood and scrubwood

*Commidendrum robustum* (St Helena gumwood) and *C. rugosum* (St Helena scrubwood) are clearly distinct species with distinct morphology and ecologies (Ashmole and Ashmole 2000). The species are known to hybridise, forming morphotypes intermediate to the parental species (Eastwood et al. 2004). Differing capitulum morphologies suggest that pollination might have a role in enforcing the species boundary. Furthermore, although there is overlap and differences across sites, there seems to be some differentiation between the two species in their main insect visitors, with moths often abundant on *C. robustum* capitula (at least at Millennium Forest), while *C. rugosum* capitula appear to attract mainly flies.

As noted in the Results, moths tend to alight on top of the long-projecting anther tubes on the *C. robustum* capitulum. In this position they are likely to accumulate pollen on their undersides and upper legs and thus effect pollination. By contrast, flies visiting *C. robustum* capitula, because of their short proboscis (ca. 1–2 mm), have to probe with their heads at the mouth of the corolla tube (i.e. below the anther tubes) in order to access nectar. In this position, they may potentially avoid the pollen and styles presented above. The *C. rugosum* capitula with their shorter anther tubes may be better suited to fly pollination, as the head of the fly (covered with short hairs and thus suitable for pollen transfer) remains at a similar level as the presented pollen and styles at the top of the short anther tubes. We therefore hypothesise that the difference in anther-tube length may be a key feature in differentiating the pollination niche.

It is possible that inter-insect behaviour reinforces the difference in fly vs. moth visitation rates, as hoverflies seem to be sensitive to interference and avoid visiting capitula occupied by moths and other larger insects. A study of wild roses visited by bumblebees and syrphids showed that the presence of a bumblebee on flowers deterred syrphids (Morse 1981). This was not due to any physical interaction (the bees ignored the flies completely), merely that the presence of a bumblebee rendered the flower unattractive to flies. We noted the same phenomenon between moths and flies with the presence of a moth on a capitulum rendering the capitulum unattractive to flies. The implication of this is that, given the high populations of moths, a capitulum attractive to moths could be wholly moth-pollinated, not because it is unattractive to flies, but because flies are excluded by the other visitors. Moths exhibit a tendency to spend relatively long times on the flowers, thus effectively stopping hoverflies from visiting the flowers by this behavioural exclusion mechanism. There is an important corollary to this, in that most native moths are nocturnal and flies are diurnal, allowing an avoidance of interference. The introduction of alien day-flying moths has potentially had a significant effect on pollination, reducing the amount of fly pollination in the mixed fly/moth pollination system of *C. rugosum*.

It should be noted that our limited study cannot rule out the possibility of moths being more common visitors to *C. rugosum* capitula at other times. Nevertheless, the almost complete failure of *C. rugosum* to attract abundant and nearly ubiquitous species of day flying moths in our study does indicate that scrubwood capitula may be less attractive to moths and, in general field observations, we failed to observe nocturnal moth activity on *C. rugosum* while finding it frequently on *C. robustum* capitula. Further studies, including nocturnal observations and observations at all seasons, would be valuable for further confirmation.

### The role of Syrphid flies as pollinators in St Helena

Many fly groups are anthophilous and contribute to pollination, but of these, the hoverflies or flower flies (Diptera: Syrphidae) are generally thought to be especially important (Larson et al. 2001), although their contribution may vary (Orford et al. 2015). Many St Helena endemic plants have small white flowers in clusters that are regularly visited by the syrphid flies, especially the endemic *Sphaerophoria beattiei* and the non-endemic, but probably native *Syritta stigmatica*, apparently as a convergent myophilous pollination syndrome. Fly pollination also extends to the St Helena Asteraceae, despite Hymenoptera being a dominant group in the pollination of Continental Asteraceae (Lane 1996). A previously-studied example of a St Helena endemic plant pollinated by these flies is *Nesohedyotis arborea* (Roxb.) Bremek. (Percy and Cronk 1997). In this, it was shown that syrphid pollination is highly effective as a pollen vector up to ca. 30 m but that this falls off rapidly to be minimal at 500 m (Suppl. material 3). While the myophilous pollination syndrome is highly effective at effecting pollination within populations and effecting gene transfer in continuous habitat, it is a relatively ineffective in fragmented anthropogenic landscapes, that would benefit from long-distance gene transfer. One exception to this is the strong-flying *Eristalis tenax* (see Conclusions section below).

### Moths as pollinators in St Helena

There are currently almost 140 moth species known in St Helena, out of which some 60 are endemic to the island (Ashmole and Ashmole 2000, Roger Key pers. comm.). Most of the moth species endemic to St Helena are in the superfamily Tineoidea, family Tineidae (currently 40 species, 28 endemic) with smaller number of endemics in the superfamilies Noctuoidea (30 species, five endemic) and Pyraloidea (20 species, nine endemic). Most moth species on the island are nocturnal, with the exception of endemic *Glyphipterix semilunaris* and introduced species *Spoladea recurvalis* and *Herpetogramma licarsisalis*. Karisch (2018) reports that *Herpetogramma licarsisalis* seems to have a clear pattern in occurrence with more individuals being observed from the end of March to mid-April compared to February and early March. Variation in emergence times could therefore be one explanation for the scarcity of moths visiting the Peak Dale *C. robustum* capitula in January 2017 when the time-lapse footage was taken.

According to Karisch (2018) there were 51 species of Lepidoptera in *C. robustum* forest habitat and 19 species in *C. rugosum* shrubland habitat. The genus *Opogona* is especially widely diversified on the island and species are present in both *Commidendrum* habitats. Another important genus that has diversified on St Helena is *Helenoscoparia*, of which one species *H. nigritalis* (day and night flying) was described as being the most numerous moth species on the Island by Wollaston (Wollaston 1879) and is still relatively common (Roger Key pers. comm.). Night-time observations with a night-enabled time-lapse camera would be required to further advance knowledge on the role of native moths on the pollination of St Helena’s endemic species, as most of the endemic species seem to be largely nocturnal. Research into nocturnal moth pollination has also been identified as a gap and a research priority in the Galapagos Archipelago (Chamorro et al. 2012).

In the Galapagos, members of the woody Asteraceae genus *Scalesia* not only have capitula reminiscent of *C. robustum* (i.e. rather globular capitula on long peduncles), they are also visited by a mixture of flies and moths (McMullen and Naranjo 1994). Partial moth pollination in St Helena's *C. robustum* may not only serve to recruit a seasonally-abundant pollination service, but also might recruit insects that disperse further than flies and that extend pollination temporally, from diurnal to nocturnal. However, Lepidoptera tend to have wide seasonal variation in abundance, being usually highly voltine, so reinforcing the need for a generalist guild of pollinators in moth-pollinated plants (Pettersson 1991). This may restrict any further specialisation towards obligate moth pollination. We have at present no information as to the relative effectiveness of moth vs. fly flower visitors as pollinators in St Helena, but such information would be useful in this regard. Further studies of endemic plant pollination on St Helena are greatly needed.

## Conclusions

### Conservation implications of pollination in *Commidendrum*

In cases where habitats are altered or fragmented, pollinator-plant mutualisms can be altered or destroyed with impacts on plant fitness and survival (Kearns et al. 1998, Fabienne Harris and Johnson 2007). There are three major plant conservation concerns: (1) that pollination failure can occur within populations leading to reproductive failure (Wilcock and Neiland 2002); (2) that habitat fragmentation and/or compromised pollination services prevent gene flow between populations and promote inbreeding and (3) pollinator and/or plant distributional changes break down pollinator-mediated species barriers and lead to interspecific gene flow, which can have both positive and negative evolutionary outcomes (Hamilton and Miller 2015). Taking the first concern, in the species considered here, seed-set within populations does not seem to be a problem. Our data show that insect visits to the capitula are numerous. Suitable native insects are still abundant enough to be effective pollinators. These have been joined by introduced flower-visiting insects, such as introduced flower-visiting moths and flies, which are now abundant.

The second concern, inter-population gene flow, is more serious. The reliance of *C. rugosum* on small insect pollinators, such as the endemic syrphid *Sphaerophoria beattiei*, that are generally not considered effective pollinators at distances of over 1 km (Percy and Cronk 1997, de Jong et al. 2005), means that many small isolated populations resulting from habitat destruction are now effectively cut off from gene flow unless supplemental conservation planting is carried out. One possible way in which populations might be connected by occasional gene flow is represented by the drone fly, *Eristalis tenax* (and to a lesser extent by the introduced Eristalinid, *Eristalinus aeneus*). Although a much less frequent visitor to the flowers than *Sphaerophoria beattiei*, *E. tenax* is a strong flier and more observations on the distribution of this species throughout the island would be useful. Its capacity for flight is considerable: specimens of *Eristalis tenax* have been taken at sea 74 km from the nearest land (Krčmar et al. 2010).

Finally, there is the issue of interspecific gene flow. A low level of interspecific gene flow has probably always occurred between the two species considered here and some apparent hybrids may be found. The overlap of pollinators, shown in this study, indicates how this may occur and highlights the importance of spatial distance, rather than pollinator specificity, in the isolation of these species. The wholesale destruction of habitat and ecological shifts, occurring over the last 500 years may have made interspecific gene flow less, or more, likely depending on specific locality. A limited amount of interspecific gene flow may have conservation benefits (Hamilton and Miller 2015) by allowing adaptive introgression (Suarez-Gonzalez et al. 2018) and so preventing genetic collapse due to inbreeding. On the other hand, under certain conditions, interspecific hybridisation can have negative impact (Todesco et al. 2016).

## Supplementary Material

1C3CF7A0-FA76-53DA-B47D-644E90B2ACDC 10.3897/BDJ.8.e52057.suppl1 Supplementary material 1 *Commidendrum rugosum* and *C. robustum* floret diagrams Data type Floret diagram Brief description Diagram of *Commidendrum rugosum* (left) and *C. robustum* (right) florets in a capitulum showing the Fibonacci patterning (divergence angle 137.4 degrees) and the daily opening of cohorts of disc flowers centripetally (alternating orange and yellow cohorts indicate successive days). Larger outer circles indicate ray flowers. File: oo_378991.pdf https://binary.pensoft.net/file/378991 Quentin Cronk, Mikko Paajanen

806EDC1F-197C-5ED0-B2A8-D948580A884F 10.3897/BDJ.8.e52057.suppl2 Supplementary material 2 Night time visitors on *Commidendrum robustum* flowers Data type Images Brief description Night-time visitors to *Commidendrum robustum* (gumwood) at Peak Dale (*Helenoscoparia scintillulalis*) and Millennium Forest (all others). Species represented, left panel: *Helenoscoparia scintillulalis*, other photographs: top row left to right: *Hypocala rostrata, Hypocala rostrata* dark form; middle row left to right: *Spodoptera littoralis*, *Trichoplusia ni*; bottom row left to right: *Herpetogramma licarsisalis*, *Spoladea recurvalis*. All photographs taken 21 February 2014 (Peak Dale) and 24 February 2014 (Millenium Forest). Note: observations on a solitary *C. rugosum* (scrubwood) at Horse Point (nearby to Millennium Forest) on the same night (24 February 2014) did not reveal any nocturnal pollinators. File: oo_380883.tiff https://binary.pensoft.net/file/380883 Mikko Paajanen

FFABDB2C-9888-5801-84CE-D9C61D3B4324 10.3897/BDJ.8.e52057.suppl3 Supplementary material 3 Female St Helena dogwood (*Nesohedyotis arborea*) fecundity in relation to distance from nearest male. Data type Diagram Brief description Effective dispersal distances of syrphid flies (*Sphaerophoria beattiei* and *Syritta stigmatica)* pollinating females of the St Helena dogwood (*Nesohedyotis arborea*) inferred from fruit set at different distances to the nearest male. The same species visit flowers of *Commidendrum robustum* and *C. rugosum*. Pollination effectiveness, measured by percentage fruit set (% fecundity), is highly efficient at short distances, but declines rapidly with increasing distance (exponential fitted curve). (Data redrawn from: Percy & Cronk, 1997). File: oo_378966.png https://binary.pensoft.net/file/378966 Diana Percy, Quentin Cronk

## Figures and Tables

**Table 1.** Regular visitors to the gumwood and scrubwood capitula. Species recorded only once are omitted from the table. Key: ** = endemic; * = native; ++ = common, + = frequent; (+) = occasional; - = absent.

**Order**	**Family**	**Scientific name**	**Common name**	***Commidendrum robustum***	*** Commidendrum *** *** rugosum ***
Lepidoptera	Pyralidae	*Spoladea recurvalis* (Fabricius, 1775)	Beet weed moth	++	-
Lepidoptera	Pyralidae	*Herpetogramma licarsisalis* (Walker, 1858)	Grass webworm	++	(+)
Lepidoptera	Lycaenidae	*Lampides boeticus* (Linnaeus, 1767)	Long-tailed blue	+	+
Diptera	Syrphidae	*Sphaerophoria beattiei* (Doesburg & Doesburg, 1977)**	Loveridge's hoverfly	++	++
Diptera	Syrphidae	*Syritta stigmatica* Loew, 1858*		+	+
Diptera	Syrphidae	*Eristalis tenax* (Linnaeus, 1758)*	Drone-fly	+	+
Hymenoptera	Apidae	*Apis mellifera* Linnaeus, 1758	Honey Bee	+	+
Diptera	Syrphidae	*Eristalinus aeneus* (Scopoli, 1763)		+	+
Coleoptera	Mordellidae	*Glipostenoda mellissiana* (Wollaston, 1870)**	Melliss's tumbling flower beetle	(+)	-
Diptera	Anthomyiidae/ Muscidae	Unidentified	"Small Black Flies"	(+)	++
Diptera	Calliphoridae/ Muscidae	*Lucilia*/*Dasyphora*	"Blue fly"	+	(+)

**Table 2.** General morphology and anthesis of the gumwood and scrubwood capitula (typical numbers).

**Characteristics**	**Gumwood**	**Scrubwood**
No. of ray florets	40	50
No. of disc florets	180	70
No. of disc florets opening per day	42	26
No. of days florets are open	4	4
No. of days capitulum receptive for pollinators	6	10

## References

[B5519749] Ashmole Philip, Ashmole Myrtle (2000). St Helena and Ascension Island: a natural history.

[B5513499] Chamorro Susana, Heleno Ruben, Olesen Jens M., McMullen Conley K., Traveset Anna (2012). Pollination patterns and plant breeding systems in the Galápagos: a review. Annals of Botany.

[B5519760] Cronk Quentin, Ninnes L (2000). The endemic flora of St Helena.

[B5513510] Cronk Q. C. B. (1989). The past and present vegetation of St Helena. Journal of Biogeography.

[B5513569] de Jong Tom J., Batenburg Judith C., Klinkhamer Peter G. L. (2005). Distance-dependent pollen limitation of seed set in some insect-pollinated dioecious plants. Acta Oecologica.

[B5513529] Eastwood A., Gibby M., Cronk Q. C.B (2004). Evolution of St Helena arborescent Astereae (Asteraceae): relationships of the genera *Commidendrum* and *Melanodendron*. Botanical Journal of the Linnean Society.

[B5513539] Edwards Joan, Smith G P, McEntee M. H. F. (2015). Long-term time-lapse video provides near complete records of floral visitation. Journal of Pollination Ecology.

[B5513559] Fabienne Harris L., Johnson Steven D. (2007). The consequences of habitat fragmentation for plant–pollinator mutualisms. International Journal of Tropical Insect Science.

[B5513549] Hamilton Jill A., Miller Joshua M. (2015). Adaptive introgression as a resource for management and genetic conservation in a changing climate. Conservation Biology.

[B5521610] Karisch Timm Darwin-Plus Project DPLUS040: Securing the future for St Helena’s endemic invertebrates: Report Lepidoptera. http://www.trust.org.sh/wp-content/uploads/2018/10/Timm-Karisch-Lepidoptera-report-2018.pdf.

[B5519632] Kearns Carol A., Inouye David W., Waser Nickolas M. (1998). Endangered mutualisms: The conservation of plant-pollinator interactions. Annual Review of Ecology and Systematics.

[B5721104] Krčmar S., Kučinić M., Durbešić P., Benović A. (2010). Insects from the middle of the Adriatic Sea. Entomologica Croatica.

[B5519796] Lambdon P (2012). Flowering plants & ferns of St Helena (Ed. A Darlow)..

[B5519842] Lane M. A., Royal Botanic Gardens Kew. (1996). Pollination biology of Compositae. Compositae: Biology & Utilization 2..

[B5519642] Larson BMH, Kevan PG, Inouye DW (2001). Flies and flowers: taxonomic diversity of anthophiles and pollinators. The Canadian Entomologist.

[B5519856] McMullen B. C.K., Naranjo S. J. (1994). Pollination of Scalesia
baurii
ssp.
hopkinsii (Asteraceae) on Pinta Island.. Noticias de Galápagos.

[B5519652] Morse Douglass H. (1981). Interactions among syrphid flies and bumblebees on flowers. Ecology.

[B5519681] Orford Katherine A., Vaughan Ian P., Memmott Jane (2015). The forgotten flies: the importance of non-syrphid Diptera as pollinators. Proceedings of the Royal Society B: Biological Sciences.

[B5519691] Percy D. M., Cronk Q. C.B. (1997). Conservation in relation to mating system in *Nesohedyotis
arborea* (Rubiaceae), a rare endemic tree from St Helena. Biological Conservation.

[B5519701] Pettersson M. W. (1991). Pollination by a guild of fluctuating moth populations: Option for unspecialization in *Silene
vulgaris*. The Journal of Ecology.

[B5519711] Suarez-Gonzalez Adriana, Lexer Christian, Cronk Quentin C. B. (2018). Adaptive introgression: a plant perspective. Biology Letters.

[B5519866] Todesco M., Pascual M. A., Owens G. L. (2016). Hybridization and extinction.. Evolutionary Applications.

[B5519662] Van der Niet Timotheüs, Peakall Rod, Johnson Steven D. (2014). Pollinator-driven ecological speciation in plants: new evidence and future perspectives. Annals of Botany.

[B5519721] Wilcock Chris, Neiland Ruth (2002). Pollination failure in plants: why it happens and when it matters. Trends in Plant Science.

[B5519876] Wollaston T. V. (1879). Notes on Lepidoptera of St. Helena, with descriptions of new species.. Annals and Magazine of Natural History.

