# Supplementary figures and images for: Moth versus fly: a preliminary study of the pollination mode of two species of endemic Asteraceae from St Helena (Commidendrum robustum and C. rugosum) and its conservation implications
Source: Biodivers Data J. 2020 May 6;8:e52057. doi: 10.3897/BDJ.8.e52057 (PMC7220971; doi:10.3897/BDJ.8.e52057)

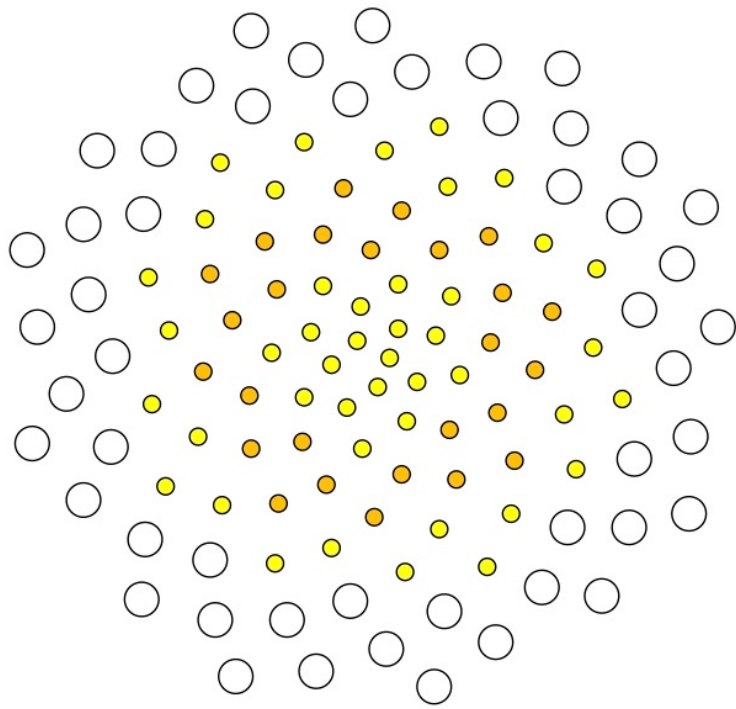

*Commidendum rugosum*

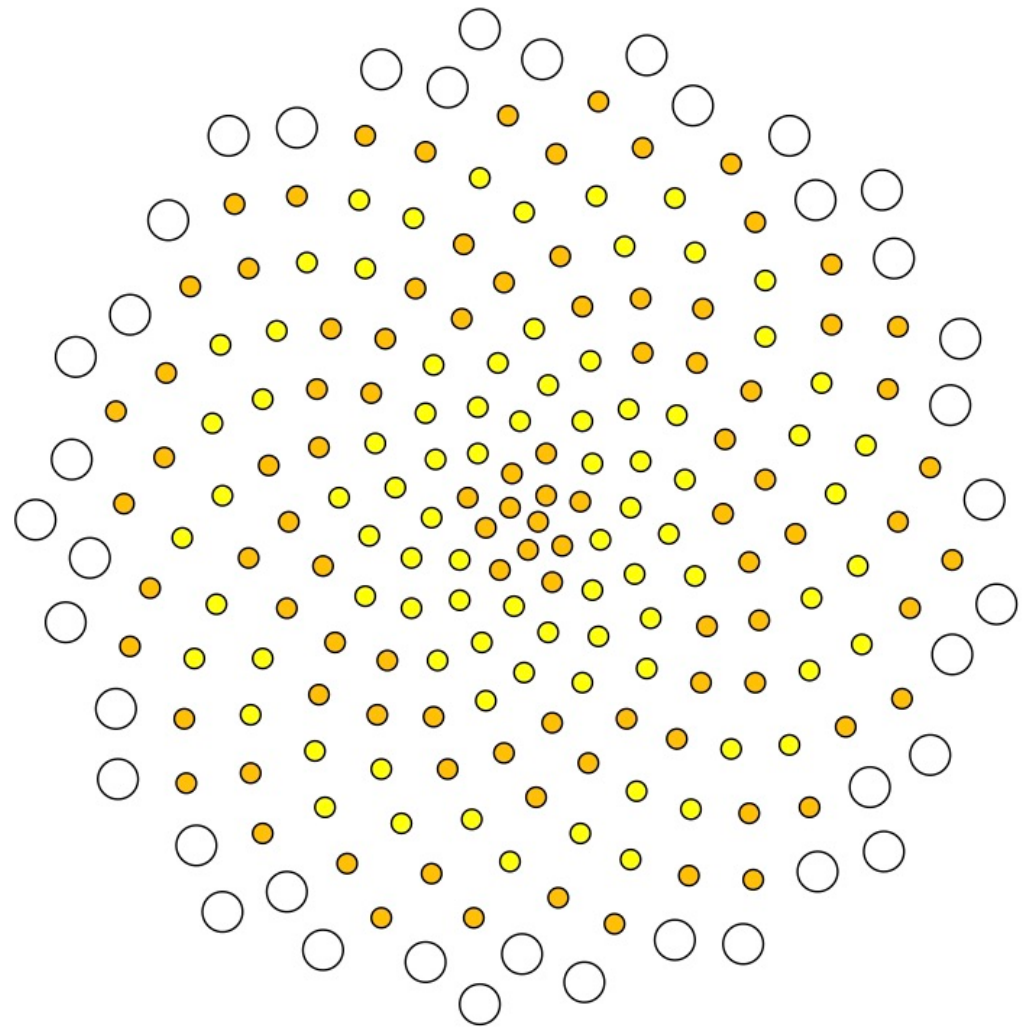

*Commidendum robustum*

Supplement: Supplementary material 1 — Commidendrum rugosum and C. robustum floret diagrams [file bdj-08-e52057-s001.pdf]

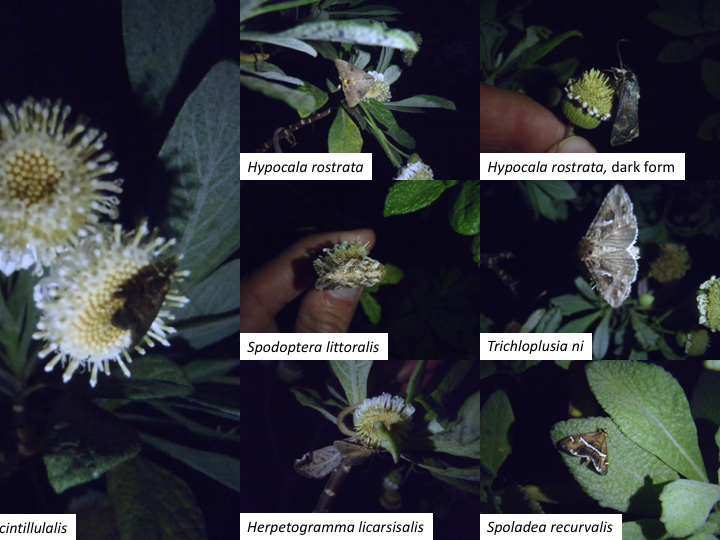

Supplement: Supplementary material 2 — Night time visitors on Commidendrum robustum flowers [file bdj-08-e52057-s002.tiff]

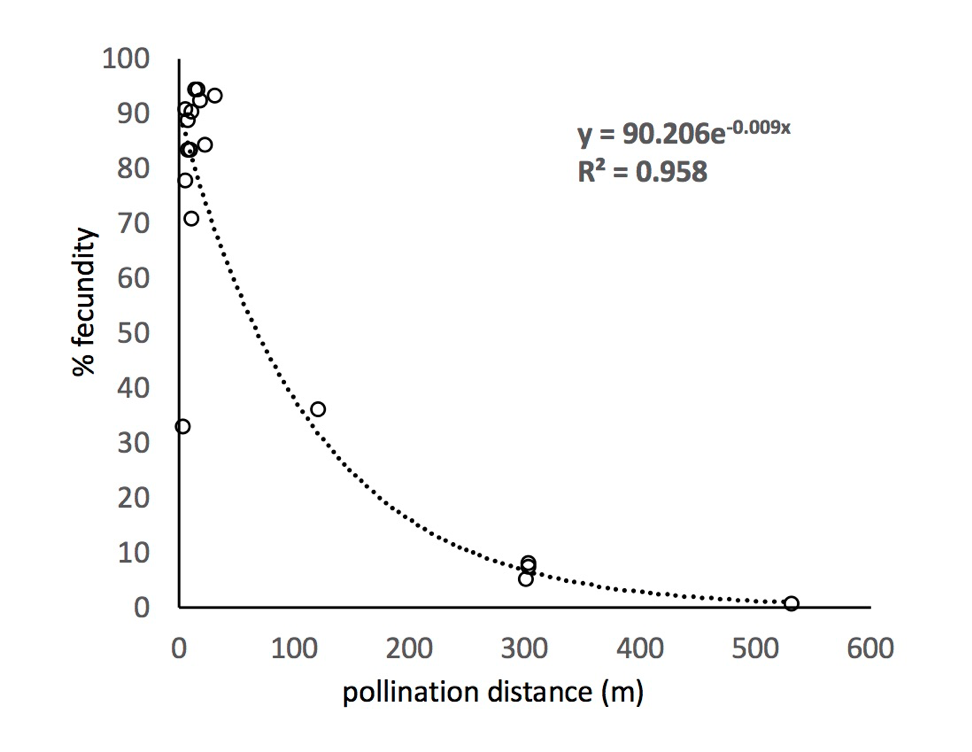

Supplement: Supplementary material 3 — Female St Helena dogwood (Nesohedyotis arborea) fecundity in relation to distance from nearest male. [file bdj-08-e52057-s003.png]
